# A Comparative Study on Safety and Efficacy of Desvenlafaxine Versus Sertraline in Depression

**DOI:** 10.7759/cureus.22717

**Published:** 2022-02-28

**Authors:** Saritha Ch, Sree Sudha, C. Gowtham Reddy, Pugazhenthan T, Krishna Sasanka KSBS, Pooja Dasari, Pradeep Battula, Nandini T, Sandeep A

**Affiliations:** 1 Psychiatry, Santhiram Medical College and General Hospital, Nandyal, IND; 2 Pharmacology, All India Institute of Medical Sciences Deoghar, Deoghar, IND; 3 Pharmacology and Therapeutics, All India Institute of Medical Sciences Raipur, Raipur, IND; 4 Otolaryngology, All India Institute of Medical Sciences Deoghar, Deoghar, IND; 5 Therapeutics, Santhiram College of Pharmacy, Nandyal, IND

**Keywords:** becks depression inventory and ham-d scales, dsm-v criteria, depression, desvenlafaxine, sertraline

## Abstract

Background

Depression is one of the most predominant mental health issues that are prevalent now. Therefore, many clinical trials were being conducted to find the safest, most effective, and tolerable anti-depressant. This study aims to compare desvenlafaxine and sertraline regarding their safety and efficacy in treating depression.

Methodology

The patients who were diagnosed with depressive disorder according to the Diagnostic and Statistical Manual of Mental Disorders (DSM-5) criteria were included in the study and were divided into two groups. The severity of depression in these patients was evaluated using Beck Depression Inventory and Hamilton depression scale (HAM-D) before and after the treatment (four weeks).

Results

About 64% of the study sample were males, and 36% were females, with 77% of the patients in the desvenlafaxine group taking 100 mg dosage and about 74% patients taking 50 mg dosage in the sertraline group. The patients in both groups showed statistically significant (p < 0.00001) improvement after using these drugs.

Conclusion

Both desvenlafaxine and sertraline showed their efficacy in treating depression by improving the clinical outcome in patients. Sertraline was marginally better in clinical results. Finally, it is advisable to carry out more randomized trials to improve the patient’s quality of life.

## Introduction

In a person's lifetime, the estimated occurrence of at least one episode of major depressive disorder is about 17% [1]. This occurrence causes psychiatrists and physicians to encounter this disorder often in their clinics. Patients suffering from depression not only experience difficulties in their social functioning but also have impaired work output [2-3]. All this has led to the recognition of depression as the fourth most leading disability globally [4]. It has been established that the primary choice in the management of depression is pharmacotherapy [5]. Antidepressants are also used to treat chronic pain, which can result in depression [6-7]. The prescriptions for treating depression mainly include second-generation antidepressants like selective serotonin reuptake inhibitors (SSRIs), serotonin and norepinephrine reuptake inhibitors (SNRIs), and other drugs that selectively target neurotransmitters [8]. SSRIs are also combined with antipsychotics to treat bipolar depression [9]. Depression is most commonly treated in three divided stages - acute, continuation, and maintenance phases [10]. From the presentation of symptoms to eliciting a clinical response comprises the acute phase. It has now been recommended to have at least six months of continuation therapy. In the maintenance phase, the psychiatrist aims to prevent the occurrence of another episode [11-12].

The improved safety and tolerability of SSRIs and SNRIs caused them to gain popularity in treating depression over the older tricyclic antidepressants [13]. Sertraline belongs to SSRIs which inhibit only serotonin reuptake. At the same time, desvenlafaxine has dual-acting properties as it can block the reuptake of serotonin and norepinephrine and belongs to SNRIs [14-15]. It has been found that there is dysregulation of serotonin and norepinephrine neurotransmitter systems in patients suffering from depression [16]. Evidence has been established regarding the safety of sertraline in the maintenance therapy of depression in various placebo-controlled trials [17].

The third SNRI that the US FDA has approved for the treatment of depression is desvenlafaxine (administered as desvenlafaxine succinate) [18]. It has been derived from SNRI venlafaxine in a salt form of its isolated major active metabolite (O-desmethylvenlafaxine). Research regarding the use of desvenlafaxine for patients suffering from major depressive disorder has been thorough. The safety, efficacy, and tolerability of desvenlafaxine have been established by many double-blind, placebo-controlled, multi-centric, and randomized trials [19]. Furthermore, the effective relieving of painful symptoms linked to major depressive disorder has been confirmed with the use of desvenlafaxine by numerous clinical trials when compared with placebo [20-22]. Unfortunately, there is a paucity in the literature for comparative studies between sertraline and desvenlafaxine, especially in rural populations. Several studies have established the safety and efficacy of these drugs compared to placebo; however, the edge of one drug over another in terms of patient's clinical response is yet to be established. The main aim of this study is to find out a better drug profile between sertraline and desvenlafaxine in relation to their safety, efficacy, and tolerability by patients suffering from a major depressive disorder.

## Materials and methods

A prospective observational study was carried out comparing the efficacy and tolerability of sertraline to desvenlafaxine in treating depression. This study was conducted at Santhiram Medical College and General Hospital, Nandyal, for a duration of six months from December 2020 to May 2021. The total sample size in this study was 81 patients. The level of depression in these patients was evaluated using Beck Depression Inventory and Hamilton depression scale (HAM-D) before and after the treatment (four weeks). The study was carried out after getting approval from the Institutional Human Ethics Committee (I.E.C./2020/080 dated 22nd December 2020), and after taking written informed consent from patients. The study was carried out from a semi-urban location, with most patients hailing from rural backgrounds. Patients enrolled in this research were divided into two groups based on a simple random sampling procedure. This study included physically healthy outpatients aged between 18 and 75 years diagnosed with depression and met the Diagnostic and Statistical Manual of Mental Disorders (DSM-5) criteria for depressive disorder. Patients hypersensitive to desvenlafaxine and sertraline or with a significant risk of suicide or suffering from depression due to general medical, neurological conditions, pregnant and breastfeeding women, and those not willing to participate in the study were excluded.

## Results

**Figure 1:**
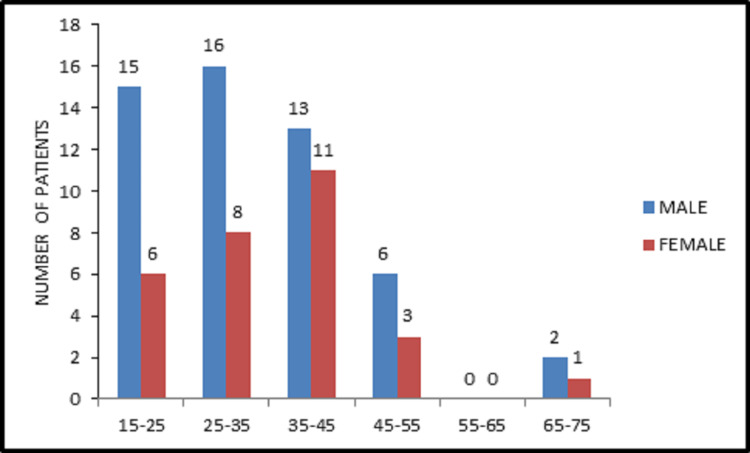
Age-wise gender distribution.

Figure 1 shows the gender distribution of the patients enrolled in the study according to their age group. Out of the 81 patients in this study, 52 were males, and 29 were females. The majority of the patients (29.6%) were found to be equally distributed in the age groups of 35-45 years and 25-35 years group. They were followed by 21 patients (26%) in the 15-25 years age group.

**Figure 2:**
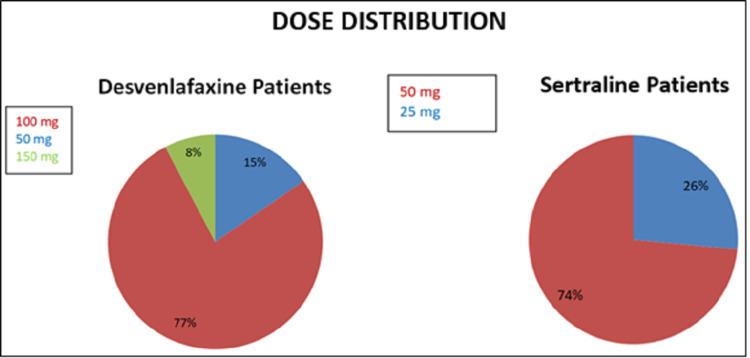
Distribution of dosage in both groups.

Figure 2 shows that the patients in the study were divided into two groups based on the dosage distribution and the prescribed drugs. In the group prescribed with desvenlafaxine, the majority (77%) of them were taking a dosage of 100 mg. On the other hand, the majority (74%) of the patients in the sertraline group were using a dosage of 50 mg.

Table 1 shows the levels of depression among the patients in the current study before and after the treatment by applying HAM-D and Beck Depression Inventory to them. In the desvenlafaxine group prior to treatment, the patients scored a mean of 17.1282 and a mean of 25.6923 on the HAM-D and Beck Depression Inventory. After being treated with desvenlafaxine, they improved their scores by getting a mean of 9.5385 and 16.0256 on the HAM-D and Beck Depression Inventory, respectively. The patients in the sertraline group scored a mean score of 17.6905 and 25.8095 before the onset of their treatment on the HAM-D and Beck Depression Inventory. However, after taking sertraline, the patients scored a mean of 8.4048 and 14.381 on these scales.

**Table 1:** Comparison of levels of depression using HAM-D and Beck Depression Inventory before and after the treatment.

Level of depression	Drug	Before treatment Mean score (SD)	After treatment Mean score (SD)	t-value	p-value
Hamilton Depression Scale	Desvenlafaxine	17.1282 (5.6391)	9.5385 (3.8241)	-10.048723	P < 0.00001
Beck Depression Inventory	Desvenlafaxine	25.6923 (7.3095)	16.0256 (4.475)	-10.415067	p < 0.00001
Hamilton Depression Scale	Sertraline	17.6905 (5.5765)	8.4048 (3.4995)	-13.972106	p < 0.00001
Beck Depression Inventory	Sertraline	25.8095 (9.4152)	14.381 (5.6264)	-10.925345	p < 0.00001

Table 2 represents the level of depression in the patients enrolled in the study before the treatment, after assigning them to two groups. Of the 42 patients in the sertraline group, 52.3% were suffering from severe depression, 28.5% with minimal depression, and 19% in the moderate category. In the 39 patients allocated to the desvenlafaxine group, 43.5% had severe depression, 30.7% in the minimal depression category, and later 25.6% had moderate depression.

**Table 2:** Severity of depression based on HAM-D score. HAM-D: Hamilton depression scale.

Treatment group (Before)	HAM-D scores			Total
	Minimal	Moderate	Severe	
Sertraline	12 (28.5%)	8 (19%)	22 (52.3%)	42 (100%)
Desvenlafaxine	12 (30.7%)	10 (25.6%)	17 (43.5%)	39 (100%)

**Figure 3:**
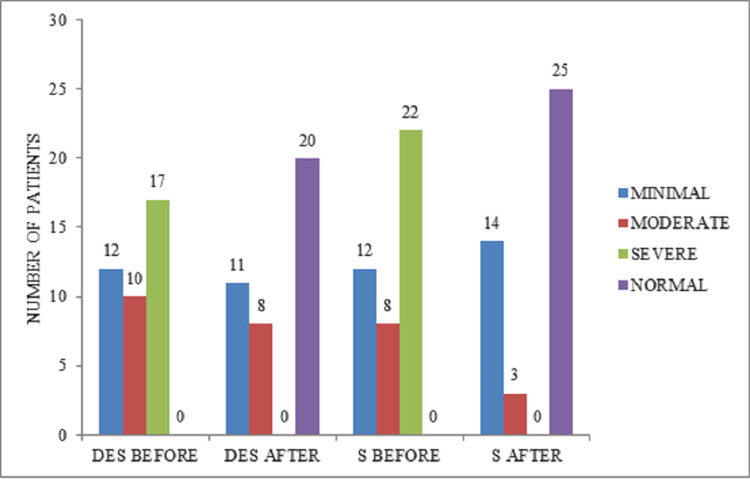
Representation of levels of depression among two groups.

Figure 3 shows the levels of depression among both groups. The levels of depression among the patients who were treated with desvenlafaxine before the treatment were 12 (39.7%), 10 (25.64%), and 17 (43.58%) with minimal depression, moderate depression, severe depression, respectively. However, the levels of depression among the patients who were treated with desvenlafaxine after the treatment were 20 (51.28%), 11 (28.20%), and 8 (20.51%) with normal, minimal depression, and moderate depression, respectively.

The levels of depression among the patients who were treated with sertraline before the treatment were 12 (29.26%), 8 (19.51%), and 22 (53.65%) with minimal depression, moderate depression, and severe depression, respectively. On the other hand, the levels of depression among the patients who were treated with sertraline after the treatment were 25 (60.97%), 14 (34.14%), and 3 (7.31%) with normal, minimal depression, and moderate depression, respectively.

## Discussion

One of the main areas in the field of research related to depression is an attempt to find an effective, tolerable antidepressant based on the patient's characteristics like tolerability, response, and minimal side effects. In the present study, a comparison was carried out between desvenlafaxine and sertraline to assess how they affect the depression of patients. About 64% (52) of the study population were males, and 36% (29) were females, indicating a slight male preponderance towards depression. This variability in the results might be due to the exclusion of gender-specific risk factors like pregnancy and breastfeeding women from this study. This was in contrast with a survey by Kessler RC et al. (1994), where 13% women versus 8% men had major depression in a twelve-month period in the United States. The authors concluded that females were approximately 1.7 times more likely to suffer from depression when compared with males [22]. However, in a systematic review carried out by Abate KH regarding the gender disparity in the prevalence of depression among the patient population, the author concluded that depression was more common among females than in males [23]. The patients enrolled in the current study were mostly (29.6%) from 35-45 and 25-35 years age groups, followed by 26% in the 15-25 years age bracket. In a placebo-controlled, randomized trial carried out by Lewis G et al. about the effectiveness of sertraline in depression, the majority (41%) of the patients belonged to the age group of 18-34 years, followed by 35-54 years (40%) [24]. This difference in the age distribution might be due to the dissimilarity between the sample size of the studies. Anyhow, studies by Bagby RM et al. [25] and Gartlehner G et al. [8] showed that there was no inﬂuence of age on clinical response. The patients in both the groups were given the required dosage of drugs based on their clinical response at the time of follow up, with the majority (77%) of them in the desvenlafaxine group having a dosage of 100 mg and about 74% of the patients in sertraline group taking 50 mg dose. These results differed from Mahajan SS et al., where they calculated the mean dose of escitalopram and desvenlafaxine given to postmenopausal women with anxiety and depression [26]. Lieberman DZ and Massey SH carried out a review about the place of desvenlafaxine in the treatment of major depression and compared its safety and efficacy with other drugs. They found that the dose of 100 mg was well tolerated and safe when compared to a placebo in an eight-week double-blind trial [27]. The disparity in the results with the current study is due to the usage of higher doses of the drug, comparison with other drugs, placebo, and greater sample size. The findings in this study regarding the mean score achieved by the patients in both the groups prior to treatment on the HAM-D and Beck Depression Inventory were similar with a mean of 17 and 25, respectively. Post-treatment, the HAM-D and Beck Depression Inventory mean in the desvenlafaxine group was 9.5 and 16, respectively. While the post-treatment sertraline group showed an improvement by scoring a mean of 8.6 and 14.8 on HAM-D and Beck Depression Inventory, respectively. From this, we can infer that the sertraline group showed a slight margin of improvement in their symptoms compared to that of the desvenlafaxine group. Lustman PJ et al., in their double-blinded, placebo-controlled, randomized trial with sertraline for prevention of depression, used the HAM-D and Beck Depression Inventory at baseline and at random in the first four months of treatment. They found the mean score at baseline on Beck Depression Inventory and HAM-D to be 21.7 ± 6.8 and 15.7 ± 4.9 in the sertraline group, respectively. After four months of treatment, the mean scores were 4.4 ± 3 and 3.3 ± 2.7 on the Beck Depression Inventory and HAM-D, respectively [28]. The variation in the mean scores of the current study and the placebo-controlled trial may be due to the larger sample size and a different study design. 

In this study, about 39 patients in the desvenlafaxine group before treatment had minimal, moderate, and severe levels of depression. After taking treatment with desvenlafaxine, they showed improvement, with 20 patients being normal followed by 11 patients with minimal depression. About 42 patients in the sertraline group had minimal, moderate, and severe levels of depression previous to their treatment. Subsequent to their treatment with sertraline, about 25 patients were normal, followed by 13 patients with minimal depression. From the above results, we can gather that patients in the sertraline group showed a better clinical response when compared to those in the desvenlafaxine group. The present study has several limitations in the form of a smaller sample size with samples collected from a single institution with results not generalizable to the whole population. In addition, the duration of observation was at baseline and then four weeks after the onset of treatment. This is a shortcoming as some patients may show better antidepressant response at 6-8 weeks after treatment. Another drawback of this study was that the adverse effects of these drugs were not monitored. Selection bias of the study subjects could be avoided by randomization, which we recommend for future studies.

## Conclusions

Both sertraline and desvenlafaxine showed efficacy in treating depression and were well-tolerated as indicated by the application of the HAM-D and Beck Depression Inventory, meeting the hypothesis given by the author. Sertraline showed a better clinical outcome when compared to desvenlafaxine in the present study. This study helps in comparing the drug one on one, unlike the previous studies where the two groups were compared and helps in adding to the current literature. However, a larger randomized comparative clinical trial is recommended to establish the superior efficacy and tolerability of these drugs.

## Figures and Tables

**Figure 4:**
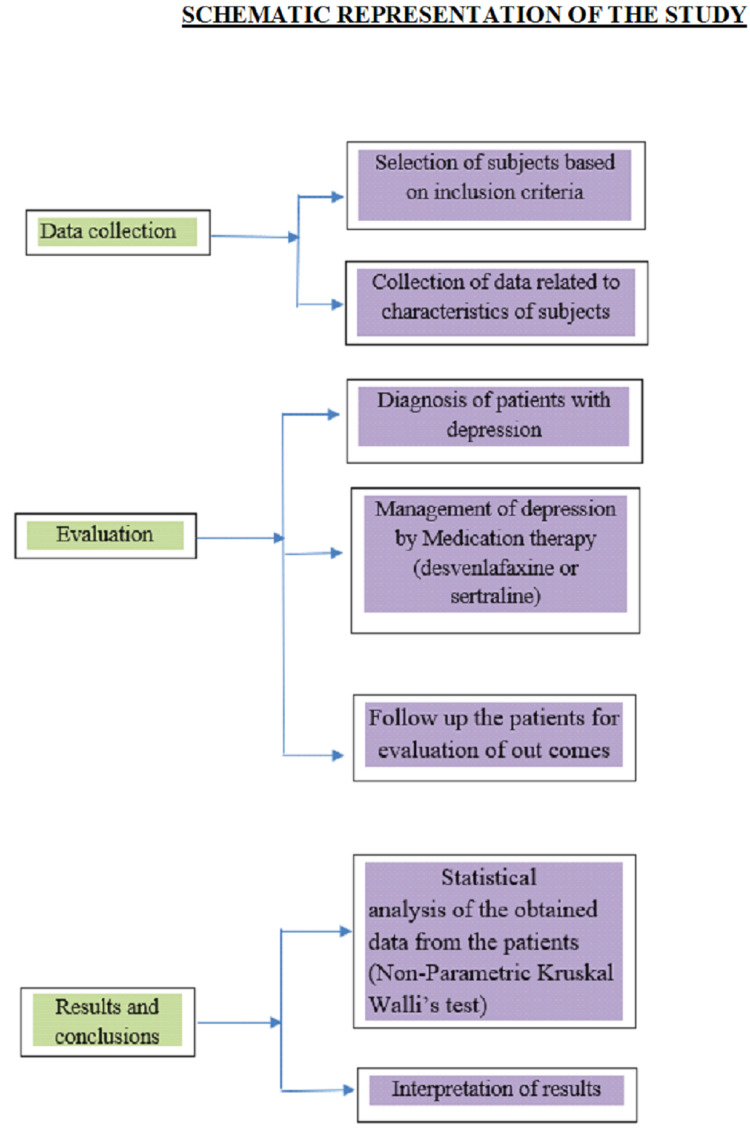
Schematic representation of the study.
